# Patients’ knowledge, attitudes, preferences regarding kidney cancer screening, and factors influencing participation intentions: a qualitative study

**DOI:** 10.3389/fonc.2026.1699073

**Published:** 2026-02-19

**Authors:** Xiao Ma, Rong Liu, Hui Zhuo, Xiang Xi

**Affiliations:** Department of Urology, The Third People’s Hospital of Chengdu, Chengdu, Sichuan, China

**Keywords:** attitudes, influencing factors, perceptions, preferences, renal cell carcinoma, screening, kidney cancer

## Abstract

**Introduction:**

Screening is a crucial method for improving the early detection rate of Kidney Cancer (KC). Numerous challenges currently exist in the promotion and implementation of KC screening. As direct participants in the screening and diagnostic process, KC patients possess a deeper understanding of the screening procedures. Clarifying patients’ attitudes and preferences toward KC screening is particularly important for optimizing screening policies.

**Methods:**

This study employed purposive sampling to select KC patients as research subjects. Using the phenomenological research method within qualitative research, semi-structured interviews were conducted to gather participants’ attitudes and preferences regarding KC screening. Data analysis was performed using Colaizzi’s seven-step method to extract themes related to KC screening.

**Results:**

This study identified 7 themes and 29 subthemes centered on three core dimensions: “Attitudes and Preferences Toward KC Screening,” “Factors Influencing Participation in KC Screening,” and “Access to Screening Information.” Recognition of screening value (including 3 sub-themes: intervention in disease progression, screening necessity for asymptomatic individuals, screening demand among high-risk populations); screening method focus dimensions and selection preferences (including 5 sub-themes: accuracy, convenience, safety, affordability, scientific validity). Facilitating factors (including 5 sub-themes: health consciousness, positive social support, risk awareness motivation, authoritative advice, and convenient screening opportunities); Barriers (including 6 sub-themes: complacency, time constraints, information resource limitations, financial burden, psychological avoidance, accuracy concerns). Information Acquisition Channels (including 4 sub-themes: healthcare provider communication, online media, interpersonal exchange, physical promotional materials); Healthcare Provider Assistance (including 3 sub-themes: process guidance, accessible information delivery, psychological counseling); Preferred Screening Promotion Channels (including 3 sub-themes: offline promotional events, online science communication, public welfare activities).

**Conclusions:**

This study clarifies renal cancer patients’ attitudes and preferences regarding renal cancer screening, as well as the factors influencing their participation in screening, providing a reference for optimizing screening strategies. The study recommends that future efforts should integrate public needs by implementing risk stratification, optimizing screening technologies, improving healthcare coverage mechanisms, simplifying service procedures, strengthening public risk education, and emphasizing psychological counseling. These measures will enhance participation rates and implementation effectiveness of renal cancer screening.

## 1 Introduction

Kidney Cancer (KC) is one of the most common malignant tumors of the urinary system (1). Globally, KC ranks as the sixth most common malignant tumor in men and the tenth in women. With the projected growth of the global population over the coming decades, its incidence is expected to continue rising, posing a serious threat to human health (2). Renal cell carcinoma (RCC) accounts for approximately 90% of all KC cases and is the most common pathological subtype of this disease (3, 4). In China, driven by the accelerating pace of population aging and shifts in residents’ lifestyles, the incidence, prevalence, and mortality rates of KC have all shown a persistent upward trend over the past few decades (5). Research predicts that the incidence and mortality rates of KC in China will continue to rise over the next decade, with approximately 130,000 new cases expected by 2030 (6). Early detection, diagnosis, and treatment are critical to improving patient survival rates and quality of life (7, 8).

Most RCC patients exhibit no obvious symptoms in the early stages. The majority are incidentally diagnosed during abdominal imaging studies conducted for other conditions, such as abdominal pain or urinary abnormalities. Approximately 35% of patients have metastatic disease at the time of diagnosis (9). By the time RCC-related clinical symptoms appear, most patients are already in advanced stages of the disease. Patients with stage I RCC detected through ultrasound or CT screening exhibit a 37% higher five-year survival rate compared to those who present only after symptoms develop (10). Timely screening of high-risk populations can effectively increase early detection rates and reduce mortality (11).

Internationally, Western countries initiated KC research earlier, but studies have primarily focused on screening methods and technologies, with limited investigation into subjective experiences such as attitudes and preferences among screened populations (12). Domestic research in China has also concentrated on the efficacy of screening technologies, such as the application of ultrasound and CT in renal cancer screening (6), while studies examining public attitudes and preferences toward renal cancer screening remain insufficient.

Currently, KC screening faces numerous challenges in its promotion and implementation. Compared to highly prevalent cancer screening programs like lung cancer screening, public awareness of KC screening remains significantly low (13). Inadequate and distorted public perceptions of KC screening lead to low willingness to participate proactively (14). Current research on the varying needs and acceptance levels of KC screening among populations with different educational backgrounds and economic statuses is limited, making it difficult to develop personalized screening recommendations.

Previous studies on attitudes toward renal cancer screening have been limited, primarily focusing on the general public to explore public awareness and willingness to accept screening (15), with insufficient attention given to the core group of renal cancer patients. Existing research from the patient perspective has mostly centered on postoperative experiences (16), lacking exploration of patients’ attitudes and preferences regarding renal cancer screening services. Optimizing KC screening protocols requires consideration of real-world clinical needs. The current lack of patient-level feedback makes it difficult to fully align with clinical practice.

As direct participants in screening and treatment processes, renal cancer patients possess intuitive and authentic insights into the necessity of screening, procedural convenience, information clarity, and potential needs. Their perspectives can reveal practical challenges and areas for improvement in screening practices, offering valuable reference for enhancing screening services (17). Therefore, gaining a deep understanding of KC patients’ attitudes toward screening, their preferences, and the factors influencing their participation holds significant practical importance for enhancing KC screening efforts and improving screening efficiency and effectiveness. This study employed semi-structured interviews as a qualitative research method to investigate the attitudes and preferences of patients with confirmed RCC toward screening for KC. It aimed to identify factors influencing their participation in screening programs, thereby providing insights for optimizing renal cell carcinoma screening protocols.

## 2 Method

### 2.1 Participants and recruitment

This study is a single-center investigation conducted at a tertiary hospital in Chengdu, Sichuan Province, China. All participants were renal cancer patients who sought treatment at the hospital’s Department of Urology. Research subjects were selected using purposive sampling, including renal cancer patients of varying ages, disease stages, treatment histories, educational levels, and residential locations to encompass diverse perspectives. Inclusion criteria: Pathologically confirmed renal cancer diagnosis (18); age ≥18 years; no language communication barriers, with the ability to accurately express genuine inner experiences; stable disease status under standardized treatment; Understand the diagnosis of their own condition, its progression, and treatment plan, and voluntarily participate in this study. Exclusion criteria: Individuals with severe mental illness or cognitive impairment preventing normal communication; Those who refuse to participate in this study. The sample size for this study was determined based on the principle of theoretical saturation in qualitative research. During formal interviews, coding and thematic extraction were conducted synchronously using Colaizzi’s seven-step method after every 3–5 completed interviews. When no new themes emerged across two consecutive rounds of analysis, and existing themes were fully validated with detailed repetition, two additional interviews were conducted. Upon confirming no new information emerged, the current sample size was deemed to have reached theoretical saturation (19).

### 2.2 Interview guide

Based on relevant literature and clinical experience (20), the research team initially developed an interview guide. Three renal cancer patients were selected for pre-interviews. Following discussions and revisions based on the pre-interview results and expert consultation recommendations, the formal interview guide was finalized. The interview guide is detailed in **Supplementary File 1**.

### 2.3 Data collection

This study employed a descriptive phenomenological approach within qualitative research. In August 2025, semi-structured interviews were conducted to gather information on renal cancer patients’ attitudes and preferences regarding renal cancer screening. Interviewers were clinical nursing staff specializing in urology with over three years of experience and holding intermediate-level professional titles or higher. One-on-one interviews were conducted in quiet, comfortable health consultation rooms, involving only the interviewer and participant. Each session lasted 60–90 minutes. Recording equipment was pre-tested, and interview outlines and documentation forms were prepared, highlighting key areas of focus. Before recording commencement, participants received full disclosure and assurances that data would be used solely for this study without compromising personal privacy. The entire interview was conducted in Mandarin Chinese. Both the interview recordings and transcribed texts retain the original Chinese expressions to ensure data authenticity. Measures to ensure interview quality included: fostering interviewee trust in the interviewer to facilitate candid expression of genuine thoughts; avoiding excessive guidance or subjective interpretation; encouraging interviewees to elaborate on topics; and promptly clarifying ambiguous statements. The entire interview is recorded synchronously, with the interviewer meticulously documenting key points of the interviewee’s responses. Simultaneously, throughout the interview process, the interviewer carefully observes the interviewee’s facial expressions, body language, and other nonverbal behaviors to gather potential insights. Following the interview, the interviewer accurately transcribes the audio recording into written text and verifies the transcription with the interviewee to ensure the authenticity and accuracy of the interview record.

### 2.4 Analysis

This study employed Colaizzi’s seven-step method (21) to analyze interview data. (1) Within 24 hours of the interview, the interviewer transcribed the audio recording into a written text sentence by sentence, preserving nonverbal information such as interjections and pauses. After transcription, the interviewer and another researcher jointly verified the transcript to ensure accuracy and completeness. (2) Research team members repeatedly review all transcribed materials to extract key statements directly relevant to the research theme, reflecting the interviewee’s authentic experiences and core perspectives. (3) Two researchers independently conduct preliminary interpretations of the extracted key statements, distilling their core meanings into meaning units and creating unique codes for each using Nvivo qualitative analysis software. If coding discrepancies arose, a third member intervened to arbitrate, ultimately establishing standardized codes. (4) The research team compared all codes, grouping those with similar or related connotations to form preliminary themes. (5) For each preliminary theme, its core meaning and manifestations were elaborated in detail, followed by the selection of 2–3 representative raw interviewee statements as supporting evidence. (6) The research team compares the connotations and explanatory content of each preliminary theme to identify overlapping categories, associative dimensions, and hierarchical relationships among themes. Based on these findings, preliminary themes are integrated and optimized to form a highly abstracted thematic framework that reveals the essence of the research phenomenon. (7) The research team returned the refined thematic framework to selected representative interviewees for validation, asking whether it accurately described their actual experiences. If discrepancies arose, researchers reanalyzed the framework from Step 1 until consensus was reached with the interviewee. This validation process was disclosed to participants during their initial interviews.

## 3 Result

### 3.1 Participants basic information

A total of 22 RCC patients were interviewed, comprising 11 males and 11 females with an average age of 58.32 years (range: 49–67 years). The cohort included patients of varying ages, disease stages, treatment histories, educational levels, and residential locations. Detailed demographic information of the interviewees is presented in **Table 1**.

**Table 1:** General information of 22 participants.

Serial number	Gender	Age (years)	Course of the disease (years)	Tumor node metastasis classification	Treatment methods	Educational level	Monthly income level	Place of residence	Family history of KC	Previous history of cancer
P01	Male	60	4	Stage II	Surgical treatment	high school degree	<3000	Urban	No	No
P02	female	50	1	Stage II	Surgical treatment	bachelor degree	3000~5000	Urban	No	No
P03	Male	63	5	Stage IV	Targeted therapy	junior high school diploma	<3000	Rural	No	Yes
P04	female	52	4	Stage I	Surgical treatment	high school degree	3000~5000	Urban	No	No
P05	Male	58	8	Stage IV	Targeted therapy combined with immunotherapy	Primary school education level	<3000	Rural	No	No
P06	female	57	3	Stage I	Surgical treatment	bachelor degree	>5000	Urban	Yes	No
P07	Male	62	7	Stage IV	Targeted therapy combined with immunotherapy	high school degree	3000~5000	Urban	No	No
P08	female	55	4	Stage I	Surgical treatment	junior high school diploma	3000~5000	Rural	No	No
P09	Male	56	6	Stage II	Surgical treatment	high school degree	<3000	Rural	No	No
P10	female	58	2	Stage I	Surgical treatment	bachelor degree	3000~5000	Urban	No	No
P11	Male	63	3	Stage III	Surgical treatment	junior high school diploma	3000~5000	Urban	No	No
P12	female	62	5	Stage II	Surgical treatment	high school degree	<3000	Rural	Yes	No
P13	Male	63	3	Stage III	Surgical treatment	Primary school education level	<3000	Rural	No	No
P14	female	49	1	Stage I	Surgical treatment	bachelor degree	>5000	Urban	No	Yes
P15	Male	57	3	Stage II	Surgical treatment	high school degree	<3000	Rural	No	No
P16	female	56	3	Stage I	Surgical treatment	bachelor degree	>5000	Urban	No	Yes
P17	Male	61	5	Stage II	Surgical treatment	junior high school diploma	3000~5000	Urban	No	No
P18	female	67	5	Stage III	Targeted therapy	high school degree	>5000	Rural	Yes	No
P19	Male	65	6	Stage IV	Targeted therapy	Primary school education level	<3000	Rural	No	No
P20	female	52	2	Stage II	Surgical treatment	bachelor degree	>5000	Urban	No	No
P21	Male	65	4	Stage III	Surgical treatment	high school degree	>5000	Urban	No	No
P22	female	52	3	Stage II	Surgical treatment	junior high school diploma	<3000	Rural	No	No

### 3.2 Participants’ attitudes and preferences regarding renal cancer screening

Two themes and eight subthemes were identified regarding participants’ attitudes and preferences toward renal cancer screening. The theme “Recognition of Screening Value” comprised three subthemes: intervention in disease progression, screening necessity for asymptomatic individuals, and screening demand among high-risk populations. The theme “Dimensions of Concern and Selection Preferences for Screening Methods” included five subthemes: accuracy, convenience, safety, affordability, and scientific validity. See **Table 2** for details.

**Table 2:** The attitudes and preferences of patients with KC towards KC screening.

Theme	Subtheme	The number of mentions	Representative quote
1. Recognition of screening value	1.1 Intervention in disease progression,	11	Early detection and treatment led to faster recovery after surgery; early screening saved my life. After being discharged from the hospital and recuperating for a while, I’ll be able to live independently. Currently, my physical strength and mental state are both excellent. (P04) If it’s detected too late, once the tumor has grown or spread, treatment becomes less effective. You might need long-term chemotherapy afterward, medical costs will skyrocket, and survival time is significantly reduced. (P10) At that time, stage I KC was detected, and it hadn’t spread yet. The surgery went well, and the recovery was good. (P10) When stage II KC was diagnosed, fortunately, it hadn’t spread. The surgery was very smooth. (P20) If I had been checked earlier and detected earlier, I definitely wouldn’t have reached stage IV. Now I have to suffer a lot more. The condition is severe, treatment has been ineffective, and physical health continues to deteriorate. (P03) My diagnosis was advanced KC. Treatment at this stage is both complicated and painful. If only it had been detected earlier, the condition wouldn’t have progressed this far, and treatment wouldn’t be so reactive. Now it’s too late to do anything about it. I truly regret it. (P05)
	1.2 Screening Necessity for asymptomatic individuals	8	No symptoms don’t mean no problem. Early detection brings peace of mind. (P01) Without any discomfort in the early stage, by the time symptoms appear, the disease has already progressed to an advanced stage. (P03)
	1.3 screening demand among high-risk populations	7	My father had cancer before. It was detected too late, and the later treatment was ineffective, causing him a lot of pain. I have a family history of cancer and need to undergo regular check-ups. (P06) I had other types of cancer before, and the risk was high. Knowing how to screen and detect early can help reduce the suffering. (P16) As people get older, they are more prone to illness. It is recommended that people aged 50 and above have regular physical examinations. (P08)
screening method, focus dimensions, and selection preferences	2.1 accuracy	19	Checking for accuracy is of the utmost importance. Failing to detect errors can delay the treatment and impose a burden on the patient. (P04) To ensure accuracy, make sure not to miss any details. If money is spent but the illness isn’t detected, it delays treatment. (P16)
	2.2 convenience	15	I’m usually quite busy, so I hope the check-up won’t take too much time. (P20) It would be better if the check process were simpler. Avoid running back and forth between departments. Ideally, you should receive your test results on the same day, eliminating the need to return to the hospital to pick them up.(P12)
	2.3 safety	10	I don’t like the examinations with wounds. I can accept non-invasive examinations like ultrasound. (P02) I’m afraid of pain. Don’t perform surgery. It’s better to avoid any wounds. (P18) I’m worried about the radiation risk of low-dose CT, so it’s better to do it less often. Excessive radiation exposure can actually harm your health. (P22)
	2.4 affordability	9	I’m from a rural area, and my income is low. The examination should not be too expensive. If it can be covered by medical insurance, that would be best. (P13) Lower costs, covered by medical insurance, with subsidies (P01)
	2.5 scientific validity	2	If there is medical evidence, don’t believe in folk remedies. (P06) I place far more trust in formal and scientific screening methods than in those hearsay approaches. (P14 )

All participants supported KC screening. They believed early detection could reduce treatment complexity and financial burden while improving quality of life (P04 mentioned, “Early detection and treatment led to faster recovery after surgery; early screening saved my life. After being discharged from the hospital and recuperating for a while, I’ll be able to live independently. Currently, my physical strength and mental state are both excellent.”; P10: “If it’s detected too late, once the tumor has grown or spread, treatment becomes less effective. You might need long-term chemotherapy afterward, medical costs will skyrocket, and survival time is significantly reduced.”). Respondents generally believed screening is highly effective, enabling early disease detection, timely treatment to gain valuable time, and preventing metastasis(P10: “At that time, stage I RCC was detected, and it hadn’t spread yet. The surgery went well, and the recovery was good.”; P20: “When stage II RCC was diagnosed, fortunately, it hadn’t spread. The surgery was very smooth.”). Participants with advanced disease (Stage IV P03, P05) deeply regretted not undergoing early screening, believing it could have reduced disease severity(P03: “If I had been checked earlier and detected earlier, I definitely wouldn’t have reached stage IV. Now I have to suffer a lot more. The condition is severe, treatment has been ineffective, and physical health continues to deteriorate.”; P05: “My diagnosis was advanced RCC. Treatment at this stage is both complicated and painful. If only it had been detected earlier, the condition wouldn’t have progressed this far, and treatment wouldn’t be so reactive. Now it’s too late to do anything about it. I truly regret it.”). Others noted that early-stage RCC often presents no obvious symptoms, making screening crucial for early detection and treatment to prevent disease progression(P01: “No symptoms don’t mean no problem. Early detection brings peace of mind. “; P03: “Without any discomfort in the early stage, by the time symptoms appear, the disease has already progressed to an advanced stage.”).

High-risk individuals have a clear need for cancer screening: participants with family histories or prior cancer diagnoses deeply understand that early detection improves treatment outcomes and reduces suffering from the disease(P06: “My father had cancer before. It was detected too late, and the later treatment was ineffective, causing him a lot of pain. I have a family history of cancer and need to undergo regular check-ups.”; P16: “I had other types of cancer before, and the risk was high. Knowing how to screen and detect early can help reduce the suffering.”). Some participants believe that as they age, their risk of developing cancer increases, necessitating regular check-ups(P08: “As people get older, they are more prone to illness. It is recommended that people aged 50 and above have regular physical examinations.”).

Participants prioritized accuracy when selecting early-stage KC screening methods, emphasizing the need to avoid missed diagnoses and misdiagnoses (P04 stressed that “screening accuracy is paramount; incorrect results delay treatment and burden the public.”; P16: “To ensure accuracy, make sure not to miss any details. If money is spent but the illness isn’t detected, it delays treatment.”). Convenience of screening methods is another key concern for respondents, who desire simple, uncomplicated processes that save time (P20 mentioned, “I’m usually quite busy, so I hope the checkup doesn’t take too long.”; P12: “It would be better if the check process were simpler. Avoid running back and forth between departments. Ideally, you should receive your test results on the same day, eliminating the need to return to the hospital to pick them up.”). Among these, participants also prioritized the safety of screening methods, generally preferring non-invasive approaches with minimal discomfort (P02 emphasized, “I dislike tests that involve wounds; I can accept non-invasive tests like ultrasound.”; P18: “I’m afraid of pain. Don’t perform surgery. It’s better to avoid any wounds.”; P22: “I’m worried about the radiation risk of low-dose CT, so it’s better to do it less often. Excessive exposure to radiation can actually harm your health.”). Financial burden was another major concern. Respondents desired reasonable costs and insurance coverage for KC screening (P13: “I’m from a rural area, and my income is low. The examination should not be too expensive. If it can be covered by medical insurance, that would be best.”; P01 hoped for “lower costs, insurance coverage, and subsidies”). Some participants emphasized the scientific validity of early-stage screening methods, preferring approaches backed by medical evidence (P06 stressed “methods with medical evidence, not folk remedies.”; P14: “I place far more trust in formal and scientific screening methods than in those hearsay approaches.”).

When selecting KC screening methods, participants preferred simple, minimally invasive, and safe approaches while also anticipating personalized and precision screening options. Urinalysis proved straightforward and easy to perform, requiring no complex procedures, and was accepted by most participants. Ultrasound scans were readily accepted due to their painless and non-invasive nature. Blood tests, requiring only a small blood sample and featuring a streamlined process, achieved high acceptance rates among participants. A minority expressed reluctance toward low-dose CT due to concerns about radiation exposure. Some participants believed risk-stratified screening strategies could enhance the targeting and effectiveness of screening efforts.

### 3.3 Factors influencing participation in renal cancer screening

Two themes and 11 subthemes were identified among the factors influencing patients’ willingness to participate. The “facilitating factors” theme comprised five subthemes: health consciousness, positive social support, risk awareness and motivation, authoritative recommendations, and convenient screening access. The “barriers” theme included six subthemes: complacency, time constraints, limited information resources, financial burden, psychological avoidance, and concerns about screening accuracy, as shown in **Table 3**.

**Table 3:** Factors influencing participation in KC screening.

Theme	Subtheme	The number of mentions	Representative quote
1. Facilitating factors	1.1 Health consciousness	15	I attach great importance to health. Therefore, I make it a point to have regular check-ups to detect any diseases early (P10)
	1.2 Positive social support	6	A friend was diagnosed with cancer, so I was reminded to have a physical examination. (P08) As I got older, I was worried about having some health problems, so my family urged me to have a check-up. (P05)
	1.3 Risk Awareness Motivation	7	There is a family history, so regular screening is necessary. (P06) I had cancer before, and the doctor advised regular check-ups. I dare not skip them. (P14)
	1.4 Authoritative advice	3	The community doctor told me that I needed to have regular check-ups to detect any problems early and receive treatment promptly (P04)
	1.5 Convenient screening opportunities	2	Our company regularly organizes physical examinations for its employees, and the abnormalities discovered during these examinations are recorded. (P02)
2. Barriers	2.1 complacency	11	At first, I thought I wouldn’t get sick since I didn’t feel any discomfort. (P18) I feel healthy and have no family history of the disease, so I shouldn’t get it. (P11)
	2.2 Time constraints	10	Work is busy, and I don’t have time to do a thorough check. (P20) The village is far from the hospital, the round trip is long, and waiting at the hospital takes too much time. I don’t have the time or energy to check. (P12)
	2.3 Information Resource Limitations	9	Previously, I wasn’t aware of the existence of KC screening and didn’t have the habit of regular physical examinations. (P15) Our family lives in the countryside, and the nearest hospital offering comprehensive physical exams is too far away. Traveling there and back is inconvenient and takes up most of the day. In the past, whenever I felt unwell, I’d just go to the township clinic to buy some medicine. I never had the habit of getting regular checkups. (P19)
	2.4 Financial burden	9	Afraid of spending money, the family’s financial situation is average. Also, afraid of uncovering any problems that might burden the child. (P13)
	2.5 Psychological Avoidance	3	I’m afraid they’ll find something wrong. The pressure is overwhelming. If they diagnose cancer, it’s hard to treat, and I’d be living in constant fear every day. (P19)
	2.6 accuracy concerns	1	I’m afraid the results won’t be accurate, wasting money and causing unnecessary anxiety. (P07)

The individual’s emphasis on health is one of the important promoting factors (P10: “I place more importance on health and I also have regular check-ups to detect diseases early.”). Positive support from the family or society also encourages participants to participate in screening (P05: “As I get older, I’m worried about having health problems, and my family urges me to have a check-up.”, P08: “A friend had cancer and reminded me to have a physical examination.”). Risk awareness brought by family history and previous cancer history will encourage the public to participate in screening (P06: “Due to family history, regular screening is necessary.”; P14: “I had cancer before, and the doctor advised regular check-ups. I dare not skip them.”). The advice from authoritative figures such as doctors, nurses, and health managers also has a promoting effect on participants (P04: “The community doctor called me to have regular physical examinations, to detect problems early and treat them promptly.” P19: “When I visited the health center earlier, the nurse explained the importance of disease screening and urged us to get regular checkups. I think we really should take this seriously.” P20: “My health management professional often sends me educational materials, explaining disease knowledge and recommending regular physicals. They even provided a detailed checkup plan, which is why I went for the tests.”). Convenient screening opportunities, such as regular physical examinations organized by the unit, will also urge the population to participate in screening (P02: “Our company regularly organizes physical examinations for its employees, and the abnormalities discovered during these examinations are recorded.”).

Time cost: Some participants are reluctant to undergo screening due to busy work schedules and lengthy hospital queues. (P20: “Too busy with work, no time to go specifically for screening.”; P12: “The village is far from the hospital, the round trip is long, and waiting at the hospital takes too much time. I don’t have the time or energy to check.”). Some participants harbor a complacent mindset, believing that the absence of symptoms means they are fine (P18: “At first, I thought I wouldn’t get sick since I didn’t feel any discomfort.”; P11: “I feel healthy and have no family history of the disease, so I shouldn’t get it.”); Participants in remote areas often missed screenings due to information gaps or resource constraints (P15: “Previously, I wasn’t aware of the existence of KC screening and didn’t have the habit of regular physical examinations.”; P19: “Our family lives in the countryside, and the nearest hospital offering comprehensive physical exams is too far away. Traveling there and back is inconvenient and takes up most of the day. In the past, whenever I felt unwell, I’d just go to the township clinic to buy some medicine. I never had the habit of getting regular checkups.”). Cost was also a factor (P13”Worried about expenses; our family’s financial situation is average. Feared a diagnosis would burden my children.”). Some participants avoided screening due to concerns about treatment difficulty after diagnosis (P19 “I’m afraid they’ll find something wrong. The pressure is overwhelming. If they diagnose cancer, it’s hard to treat, and I’d be living in constant fear every day.”); a small number also worried about accuracy (P07: “I’m afraid the results won’t be accurate, wasting money and causing unnecessary anxiety.”).

### 3.4 Obtain information on renal cancer screening

The information acquisition dimension for renal cancer screening yielded 3 themes and 10 subthemes. The “Information Acquisition Channels” theme comprised 4 sub-themes: healthcare provider notification, online media, interpersonal communication, and physical promotional materials. The “Healthcare Provider Assistance” theme included 3 sub-themes: procedural guidance, information delivery in accessible formats, and psychological counseling. The “Preferred Screening Promotion Channels” theme contained 3 sub-themes: offline promotional events, online science communication, and public welfare activities. Detailed findings are presented in **Table 4**.

**Table 4:** Information on KC screening.

Theme	Subtheme	The number of mentions	Representative quote
1. Information Acquisition Channels	1.1 Healthcare Provider Communication	7	I have a history of cancer. The doctor advised me to have regular check-ups. (P14) The doctor suggested that I should have regular follow-up visits and undergo a physical examination every year. (P16)
	1.2 online media	7	Follow the health official account, and you will receive scientific information. (P02) Learned about it by watching science-related videos on TikTok. (P22) Got to know it by watching health programs on local TV stations. (P07)
	1.3 interpersonal exchange	5	A friend mentioned it during our chat. He said someone close to him was diagnosed with cancer through screening, recovered well after timely treatment, and reminded me to get regular screenings too. That’s when I started taking it seriously. (P08) I heard it from my family. My cousin said his friend was diagnosed with cancer during a physical exam, so he told me not to ignore it and to get checked myself. (P03)
	1.4 Physical promotional materials	4	I saw community bulletin boards urging seniors to prioritize health and undergo regular check-ups. (P17) I used to read the newspaper, which had a health knowledge column that shared health tips. (P21)
2. Healthcare Provider Assistance	2.1 Process Guidance	12	It would be great if we could make an online appointment for that time. Then we won’t have to wait in line when we arrive. (P20) Tell me in advance the precautions and procedures for the check-up. Don’t make me waste my time coming here. The medical staff can guide me. (P04)
	2.2 accessible information delivery	10	I hope that when communicating with me, the doctor can explain in plain language instead of using technical terms. (P01) I hope the doctor can explain and promote in an easy-to-understand way, and can make more use of image displays, video playback, etc., so that we can understand better. (P05)
	2.3 Psychological Counseling	8	The doctor should tell me more, be more patient, and spend more time communicating with us, so that I can have a better understanding of the situation and relieve my anxiety. (P21)
3. Preferred Screening Promotion Channels	3.1 offline promotional events	14	Community doctors can come to the community to conduct publicity and have face-to-face communication. (P13) Post posters at the community entrance. We can look at them more often in our daily lives. (P11)
	3.2 online science communication	10	More authoritative science popularization videos can be broadcast online to help us understand medical knowledge. (P06) Disease science popularization short videos can be played online, and science popularization articles can be published on the official account. (P14)
	3.3 Public Welfare Activities	9	Free screenings can be conducted in the community. (P13) They can offer free clinics. I can ask doctors questions directly, and they answer on the spot—much more convenient than going to the hospital myself. (P09)

This study indicates that primary sources of information regarding KC screening include: healthcare professionals informing patients (P14 and P16 both mention “I have a history of cancer, and my doctor told me to get regular checkups.”), online media such as health-related public accounts, television stations, and medical science popularization in journals (P02: “Followed health public accounts,” P22: “Learned from science popularization videos.” P07: “Got to know it by watching health programs on local TV stations.”), conversations with friends and reminders from family members (P08: “A friend mentioned it during our chat. He said someone close to him was diagnosed with cancer through screening, recovered well after timely treatment, and reminded me to get regular screenings too. That’s when I started taking it seriously.”; P03: “I heard it from my family. My cousin said his friend was diagnosed with cancer during a physical exam, so he told me not to ignore it and to get checked myself.”), and newspaper or community publicity (P17: “I saw community bulletin boards urging seniors to prioritize health and undergo regular check-ups.”; P21: “I used to read the newspaper, which had a health knowledge column that shared health tips.”).

This study found that respondents generally desired assistance and support from healthcare providers, expecting guidance during screening to simplify the examination process and streamline procedures (P20: “Being able to book appointments online so there’s no waiting in line upon arrival.”; P04: “Tell me in advance the precautions and procedures for the check-up. Don’t make me waste my time coming here. The medical staff can guide me.”). Some respondents expressed a desire for lay language explanations of results and treatment plans during early-stage KC screening, avoiding technical jargon and ensuring clear communication (P01: “I hope that when communicating with me, the doctor can explain in plain language instead of using technical terms.”; P05: “I hope the doctor can explain and promote in an easy-to-understand way, and can make more use of image displays, video playback, etc., so that we can understand better.”).Some participants also expressed a desire for healthcare providers to offer psychological support. (P21: “The doctor should tell me more, be more patient, and spend more time communicating with us, so that I can have a better understanding of the situation and relieve my anxiety.”).

Finally, respondents indicated the need to raise awareness about the importance of KC screening, suggesting the following channels: offline promotional activities (P13: “community doctors visiting neighborhoods for outreach.”; P11: “posting informational posters at neighborhood entrances.”); Online science communication via public accounts, short videos, and health programs (e.g., P06: “Broadcast educational short videos.”; P14: “Share disease-related science videos and public account articles online.”); Additionally, regular targeted initiatives such as free community screenings and hospital outreach clinics (P13: “Free community screenings.”; P09: “They can offer free clinics. I can ask doctors questions directly, and they answer on the spot—much more convenient than going to the hospital myself.”) could be organized to encourage greater public participation in KC screening and improve prognosis.

## 4 Discussion

### 4.1 Participants’ attitudes towards KC screening and their preferences for screening methods

The findings of this study indicate that all participants support early screening. They believe that early detection provides more treatment options, thereby improving survival rates and quality of life. Some participants diagnosed at an advanced stage expressed regret over missing the opportunity for early screening. An online survey of the British public revealed that most participants would be willing to undergo any form of KC screening, including urine tests, blood tests, ultrasound, or low-dose CT scans. The core reason is the public’s strong positive attitude toward screening, coupled with a widespread belief that the benefits of early cancer detection far outweigh any potential harms associated with screening (20). A nationwide survey in the United States also revealed that 87% of respondents believe routine cancer screening is a good idea and that early detection can save lives. Even though some respondents had experienced false-positive screening results, the risk of false positives or unnecessary treatments did not diminish their support for screening (22). Previous relevant findings were primarily derived from studies in the general population. This study is the first to validate the applicability of these findings in a renal cell carcinoma patient cohort, providing more comprehensive evidence-based support for optimizing renal cancer screening protocols. We also found that some patients diagnosed at an advanced stage expressed regret over missed opportunities for early screening, further underscoring the importance of early detection from the perspective of patients’ real-world experiences.

Participants prioritized accuracy when selecting renal cancer screening methods, directly reflecting the disease’s characteristic of subtle early symptoms and high misdiagnosis rates. This underscores the clinical need to optimize the sensitivity and specificity of detection methods to ensure screening technology reliability (23). Previous cancer screening studies in chronic kidney disease patients have shown (24) that patients place high importance on the reliability and effectiveness of screening methods when making choices, aiming to avoid delayed diagnosis or overtreatment due to inaccurate results. This principle equally applies to renal cancer screening scenarios. Additionally, a survey on the general public’s attitudes toward KC screening in the UK also indicated that the reliability of results is a core dimension for participants in evaluating the value of screening methods. This aligns closely with the preference observed in this study, where participants prioritized accuracy as the primary concern for screening methods (15).

Accessibility and convenience of screening methods represent critical practical considerations, requiring evaluation of out-of-pocket costs, proximity of testing locations, appointment wait times, examination duration, and comfort levels. Participants prefer screening methods that are streamlined, safe, non-invasive, and minimally painful, showing high acceptance for urine tests, blood tests, and ultrasound scans. Low-dose CT is an important screening method, but some participants refuse it due to radiation fears. This finding suggests that screening method selection must balance “detection benefits” with “public acceptability,” prioritizing the promotion of non-invasive, convenient screening technologies. Previous surveys on renal cancer screening attitudes among individuals aged 45–77 confirmed that the public demonstrates higher acceptance of non-invasive, convenient screening methods. Among these, urine testing received the highest willingness to undergo screening, significantly surpassing invasive or relatively complex procedures such as ultrasound or low-dose CT alone. Concurrently, the study revealed that the perceived “burden/inconvenience” of screening methods is a key factor influencing public willingness to participate (20). This study further validated this perspective in renal cell carcinoma patients, while also finding that patients consider factors such as accuracy and financial burden when selecting renal cancer screening methods.

Financial burden is another key concern. Rural participants particularly emphasized the need for “reasonable costs and insurance coverage.” Inadequacies in the current healthcare system’s screening coverage may lead high-risk individuals with limited financial means to forgo screening due to unaffordability, exacerbating health inequalities (25). This highlights the need for healthcare policy interventions to enhance screening affordability, such as including KC screening in insurance coverage, providing subsidies for low-income groups, and organizing public welfare screening campaigns to reduce participants’ financial concerns at the institutional level. Additionally, participants expressed a need for “precision screening,” seeking to determine screening necessity based on individual cancer risk—aligning with the “risk-oriented” principle in modern preventive medicine (26). Currently, experts advocate for risk stratification among populations to enhance detection rates, reduce resource wastage, and mitigate the issue of overdiagnosis (27). This approach has also gained recognition in screening practices for other cancers. The UK colorectal cancer screening community generally embraces the introduction of risk stratification strategies, acknowledging their potential to significantly enhance screening benefits while reducing potential harms. However, there is currently no unified consensus on the “optimal risk stratification scheme.” (28). This limitation also exists in the field of renal cancer screening, providing a clear direction for subsequent research to optimize precision screening strategies.

### 4.2 The factors influencing participants’ participation in KC screening

This study identified the primary factors promoting participants’ participation in KC screening as health consciousness, positive social support, risk awareness motivation, authoritative recommendations, and convenient screening opportunities. Regarding risk awareness, family history and prior cancer history emerged as screening drivers, consistent with previous research indicating that “personal and family medical histories are significant predictors of cancer screening” (29). Regarding external reminders, workplace health screenings provide low-cost access to screening, while personal experiences of cancer among acquaintances challenge misconceptions. Both significantly mobilize low-risk awareness groups, suggesting that promoting KC screening requires emphasizing organizational support and the influence of social networks. Additionally, personal and family emphasis on health also promotes screening. Family reminders and encouragement not only strengthen individual health awareness but also provide emotional and practical support for screening behavior. This is particularly true for groups hesitant about independent decision-making, where family support significantly boosts confidence in participating in screening. Authoritative recommendations and convenient screening opportunities are also key factors influencing participants’ engagement in KC screening, a conclusion corroborated by screening studies for other cancers. A colorectal cancer screening study among low-income populations confirmed that patients’ trust in their primary care providers significantly drove screening completion, whereas overall trust in physicians showed no significant association with screening completion rates (30). This indicates that when promoting cancer screening in the future, it is essential not only to rely on the medical community as the core implementing body but also to strengthen communication with participants and conduct targeted education. This approach helps build stable trust relationships between participants and doctors, thereby increasing screening participation rates. Additionally, a study found that establishing comprehensive mobile screening sites in communities—extending breast, colorectal, and other cancer screening services from hospitals to residents’ doorsteps—significantly reduces transportation and time costs, effectively boosting screening participation rates. Furthermore, forming multidisciplinary teams that integrate healthcare professionals with genetic counselors, IT specialists, and other experts to provide “one-stop” services—including screening, health education, and counseling—ensures both the professionalism and comprehensiveness of services. This approach maximizes participant benefits and indirectly strengthens the practical effectiveness of screening programs (31). Another study also confirmed that participants who received a combination package containing low-literacy educational materials and self-sampling tools were significantly more likely to undergo both cancer screenings compared to those receiving standard care (32).

Some participants perceived their risk of developing RCC as low, a cognitive bias linked to the disease’s characteristic lack of symptoms in its early stages (33). This bias was particularly pronounced among non-high-risk individuals, suggesting that relying solely on participants’ self-assessment of risk may lead to missed screenings in the population (23). This perception also stems from participants’ insufficient understanding of renal cancer etiology. Consequently, targeted health education initiatives are needed to help the public develop scientifically informed risk assessment awareness, rather than relying solely on subjective feelings to determine screening necessity (34). Widespread anxiety about cancer constitutes a significant psychological barrier to screening refusal. Participants’ anxiety stems not only from fear of the disease itself but also from concerns about treatment costs, family responsibilities, and uncertain prognosis, fostering avoidance behavior (35). This anxiety may intensify among participants with poorer economic conditions or heavier family burdens, leading them to avoid screening to temporarily alleviate psychological pressure. This phenomenon highlights participants’ need for psychological and social support. Emphasizing only the medical value of screening while neglecting psychological counseling may intensify participants’ resistance (36). Therefore, when promoting screening, psychological support must be provided concurrently to reduce public fear of “detecting problems” and reinforce the positive expectation that “early detection enables early cure.”

### 4.3 Information acquisition channels and assistance requests

The public accesses information about early-stage KC screening through diverse channels, with physicians serving as the primary and most trusted source. The internet serves as the primary channel for participants to actively search for information, but the quality of information varies greatly, and participants have a limited ability to discern its validity. Experiences shared by family and friends exert some influence but may also disseminate misinformation. Traditional media and community outreach remain effective for certain segments of the population. Participants universally express a need for authoritative, accessible, and tailored screening information. In the field of cancer screening, doctors often serve as one of the primary channels through which the public accesses screening information. In previous studies, all healthcare professional-led educational interventions targeting underserved populations in rural and remote areas who did not undergo timely cervical cancer screening have increased screening uptake rates and related knowledge levels. These interventions also enhanced participants’ screening-related self-efficacy and awareness (37).

Pre-screening anxiety is common, leading respondents to seek healthcare communication and emotional support. This includes explanations of screening necessity, procedures, potential outcomes, and their significance, and associated risks. They desire compassionate, clear, and timely results delivery, along with convenient pre- and post-screening consultation channels to easily access healthcare professionals for answers. Previous studies on colonoscopy screening programs targeting Hispanic men have confirmed that participants generally exhibit insufficient knowledge about screening information and fear of the screening process, while expressing a strong desire for clear screening-related information and accessible consultation channels. This aligns with the findings of this study, where respondents expressed expectations for healthcare providers to offer communication and emotional support regarding screening (38).

This study recommends promoting KC screening knowledge through accessible language and multiple channels, emphasizing the value of early screening, clarifying misconceptions, and providing clear guidance. Content must be accessible and understandable. Tailored promotional materials for different demographics should enhance the campaign’s relevance. Leveraging success stories and social influence can also boost public confidence in and willingness to participate in screening.

This study suggests that attention should be focused on high-risk populations, such as those with a family history, smoking history, or specific occupational exposures, through targeted outreach and the provision of convenient screening pathways (39). Foster supportive environments where companies assist employees in undergoing health screenings. Communities should provide accessible services using risk stratification to offer personalized screening recommendations. Strengthen public communication by thoroughly explaining screening objectives, methodologies, and limitations. Establish robust post-screening follow-up mechanisms to ensure timely management of abnormal results. Monitor technological advancements and timely adopt new risk stratification techniques and screening methods to enhance screening efficiency and accuracy (40).

### 4.4 Strengths and limitations

This study focuses on multiple aspects, including patients’ cognition, attitudes, preferences, and factors influencing their willingness to participate in renal cancer screening. Employing semi-structured interviews within a qualitative research framework, it delves deeply into patients’ genuine thoughts and underlying needs. This approach effectively addresses the limitations of previous quantitative studies in exploring patients’ subjective experiences and latent attitudes, providing qualitative research evidence to support the optimization of renal cancer screening protocols. However, this study has limitations: all data originate from a single healthcare system, resulting in a certain degree of homogeneity in patients’ medical environments and screening experiences, which may reduce the applicability of findings across different healthcare systems or geographic populations. Although the sample size meets the normative requirements for qualitative research, it remains relatively limited given the overall heterogeneity of the renal cancer patient population. While renal cell carcinoma constitutes the predominant subtype of renal cancer and clinical screening approaches exhibit high consistency across different pathological subtypes, this study did not include patients with the rare non-renal cell carcinoma subtypes, thus failing to fully encompass the relevant needs of all renal cancer subtype patients.

## 5 Conclusion

This study thoroughly investigated the factors influencing renal cancer patients’ cognition, attitudes, preferences, and willingness to participate in renal cancer screening. Results indicate that all surveyed patients support renal cancer screening. When selecting screening methods, patients prioritize test accuracy while preferring procedures that are convenient, safe, non-invasive, and minimally painful. Additionally, financial burden significantly influences screening decisions. Results indicate that core factors promoting patient participation in KC screening include health needs, social support, risk awareness, authoritative recommendations, and screening opportunities; conversely, cognitive biases and psychological anxiety represent major barriers. Regarding information acquisition, physicians are the most trusted source of screening information for patients. The quality of information disseminated online varies greatly, and patients urgently need authoritative yet accessible science communication content. This study also recommends implementing “targeted screening” for high-risk groups through population risk stratification to reduce overdiagnosis. For future research, it is suggested to conduct multicenter studies covering different regions and healthcare systems to enhance sample representativeness and to further explore the attitudes and needs of patients with different renal cell carcinoma subtypes toward screening. For practical implementation, future efforts should optimize screening technologies, improve healthcare coverage mechanisms, and streamline service processes in alignment with public needs. Concurrently, enhanced public risk education should provide authoritative, accessible science communication, while prioritizing psychological support to comprehensively boost participation rates and implementation effectiveness of renal cancer screening, ultimately improving overall patient outcomes.

## Data Availability

The original contributions presented in the study are included in the article/**Supplementary Material**. Further inquiries can be directed to the corresponding author.
