# Supplementary material for: Patients’ knowledge, attitudes, preferences regarding kidney cancer screening, and factors influencing participation intentions: a qualitative study
Source: Front Oncol. 2026 Feb 19;16:1699073. doi: 10.3389/fonc.2026.1699073 (PMC12960160; doi:10.3389/fonc.2026.1699073)
Supplement: Supplementary file 1 [file Table1.docx]

Interview Guide for Assessing Patients' Knowledge, Attitudes, Preferences, and Factors Influencing Participation Intent in Renal Cancer Screening

Please give me some of your basic information: Age, disease duration, disease stage, treatment methods, educational level, monthly income, place of residence, family history of kidney cancer, and prior history of cancer.

**1. Key Questions**

Do you support kidney cancer screening? Why?

When choosing a screening method, what aspects concern you most? Which screening method do you prefer? (Before asking respondents the question “Which screening method do you prefer?”, interviewers will briefly explain the operational procedures and applicable scenarios of commonly used clinical kidney cancer screening methods in an objective, neutral manner using plain language. The entire explanation process strictly adheres to principles of non-bias and non-suggestiveness. Only after ensuring respondents fully understand the key information about different screening methods will they be asked the question.)

What factors do you believe primarily influence your decision to undergo kidney cancer screening?

What factors encourage your participation, and what factors hinder it?

Please describe how you learned about kidney cancer screening.

**2. Secondary Concerns**

For patients diagnosed through renal cancer screening, we directly ask: “Before undergoing renal cancer screening, did you have any concerns or worries?” What specifically?

For patients who sought medical attention due to clinical symptoms, we adjust the question context to: “Before visiting the doctor for physical discomfort and undergoing related examinations, did you have any concerns or worries?” What specifically?

For patients who underwent routine examinations due to other medical histories or family histories and incidentally discovered renal lesions, we used the question: “Before each renal-related examination, did you have any concerns or worries?” What specifically?

What kind of assistance and support would you like healthcare providers to offer during the kidney cancer screening process?

**3. Possible Follow-Up Questions**

If you were to recommend kidney cancer screening methods to others, what factors would guide your recommendation?

What avenues do you believe could raise greater awareness about the importance of kidney cancer screening?

Thanks a lot.
